# Multiple mycotoxins associated with maize (*Zea mays* L.) grains harvested from subsistence farmers’ fields in southwestern Ethiopia

**DOI:** 10.1007/s12550-024-00536-3

**Published:** 2024-05-02

**Authors:** Birhane Atnafu, Chemeda Abedeta Garbaba, Fikre Lemessa, Quirico Migheli, Michael Sulyok, Alemayehu Chala

**Affiliations:** 1 Department of Horticulture and Plant Sciences, Jimma University, P.O. Box 307, Jimma, Ethiopia; 2 Department of Plant Sciences, Bule Hora University, Bule Hora, P.O. Box 144, Hagere Mariam, Ethiopia; 3 Dipartimento di Agraria and Nucleo di Ricercasulla Desertificazione (NRD), Università degli Studi di Sassari, Viale Italia 39A, 07100 Sassari, Italy; 4 University of Natural Resources and Life Sciences, Vienna, Austria; 5 Department of Agrobiotechnology (IFA-Tulln), Institute of Bioanalytics and Agro-Metabolomics, Konrad Lorenzstr. 20, A-3430 Tulln, Austria; 6 School of Plant and Horticultural Sciences, Hawassa University, P.O. Box 5, Hawassa, Ethiopia

**Keywords:** Chromatography, Grain contamination, LC–MS/MS, Toxigenic fungi, Toxins

## Abstract

Fifty-four maize grain samples freshly harvested from subsistence farmers’ fields in southwestern Ethiopia were analyzed for multiple mycotoxins using liquid chromatography-tandem mass spectrometric (LC-MS/MS) method following extraction by acetonitrile/water/acetic acid on a rotary shaker. The grain samples were contaminated with a total of 164 metabolites, of which *Fusarium* and *Penicillium* metabolites were the most prevalent accounting for 27 and 30%, respectively. All the major mycotoxins and derivatives except one (citrinin) were of *Fusarium* origin. Zearalenone was the most frequent major mycotoxin occurring in 74% of the samples at concentrations of 0.32–1310 µg/kg. It was followed by nivalenol (63%), zearalenone-sulfate (44%), and fumonisin B1 (41%). Nivalenol, nivalenol glucoside, and fusarenon-X were detected at unusually high levels of 8–1700 µg/kg, 21–184 µg/kg, and 33–149 µg/kg, respectively. Deoxynivalenol and DON-3 glucoside contaminated 32% of the samples, each at levels of 15.9–5140 µg/kg and 10–583 µg/kg, respectively. Moniliformin and W493B occurred in 96 and 22% samples at levels of 3.27–4410 µg/kg and 3–652 µg/kg, respectively. Fumonisins were also detected in the samples at levels of 9–6770 µg/kg (B1), 16–1830 µg/kg (B2), 9.5–808 µg/kg (B3), and 1.3–128 µg/kg (A1). This study confirmed the presence of an array of mycotoxins contaminating maize grains right from the field. The effect of the co-occurring mycotoxins on consumers’ health should be investigated along with that of the newly emerging ones. Results of the current study call for application of pre-harvest mycotoxin mitigation strategies to safeguard maize-based food and feed.

## Introduction

Maize (*Zea mays* L.) is the second most important cereal crop in Ethiopia next to teff (*Eragrostis tef* [Zucc.] Trotter), cultivated by more than 10 million smallholder farmers (CSA 2021). It is mainly used for domestic household consumption (88%), both as green and dry grain and the rest as animal feed and for sale in the local market (Abate et al. 2015). However, the crop is constrained by different biotic and abiotic factors of which plant pathogens are the major biotic constraints (Tolera et al. 2018).

Maize grains are highly susceptible to toxigenic fungi contamination at all stages of the value chain (from farm to fork) (Geary et al. 2016). *Aspergillus, Fusarium*, and *Penicillium* are major fungal genera commonly reported to colonize maize and maize products leading considerably to both quantitative and qualitative losses (Wu et al. 2014). Mycotoxins are secondary metabolites produced by fungi that can contaminate commodities (Leite et al. 2021). As reviewed by Chilaka et al. (2017), about 25% of food crops worldwide, including maize and maize-based products, are at risk to contamination by mycotoxins, which are responsible for considerable loss of the produce. So far, more than 400 mycotoxins are known to occur (Berthiller et al. 2007), varying from region to region depending on the prevailing climatic conditions (Medina et al. 2015), agronomic practices (Borras-Vallverdu et al. 2022), genetic factors, fungal activity, and storage conditions (Ferrigo et al. 2016). As such, they pose serious health risks to consumers (Udomkun et al. 2017; Sarrocco and Vannacci 2018). As a result, a few of these mycotoxins are regulated by several national, regional, and international governing bodies, including the European Commission (EC 2023).

Different studies have revealed the occurrence of toxigenic fungi and associated mycotoxins in maize grain in Ethiopia (Ayalew 2010; Dubale et al. 2014; Garba et al. 2018a b; Getachew et al. 2018; Worku et al. 2019; Yilma et al. 2019; Mohammed et al. 2022). However, these studies focused on stored maize, while fresh harvests were largely ignored. Therefore, the current study was carried out to assess mycotoxin contamination of maize sampled from subsistence farmers’ fields. To the best of our knowledge, this is the first comprehensive study of multi-mycotoxins in freshly harvested maize grown by subsistence farmers in Ethiopia.

## Materials and methods

### Description of the study area

**Fig. 1:**
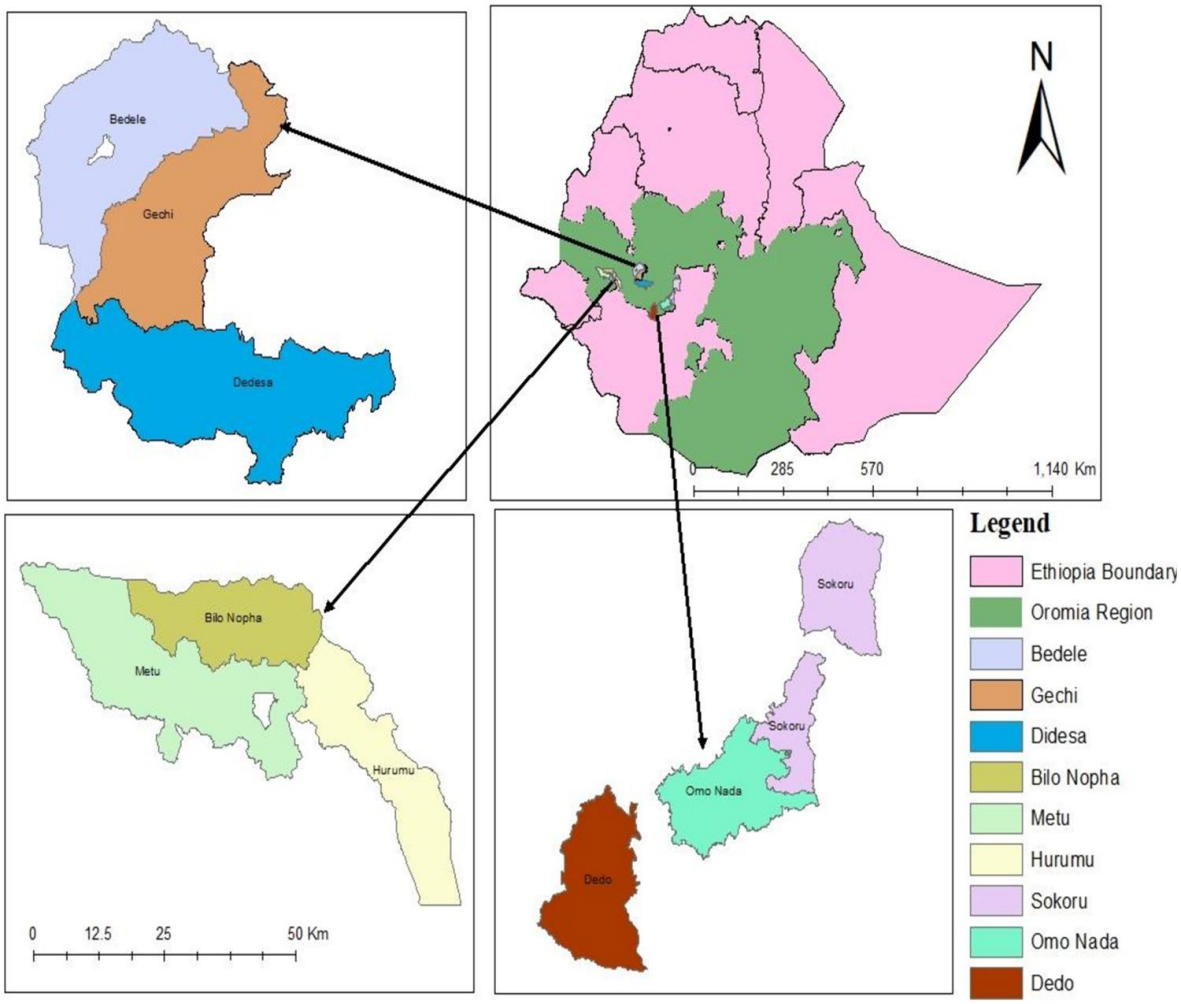
Map of survey areas in southwestern Ethiopia

Fifty-four maize grain samples were collected from farmers’ fields across three administrative zones and nine districts of southwestern part of Ethiopia (Fig. 1 and Table 1) at harvest during November to December of the 2020/21 cropping season. The study areas were selected as they are important maize-producing areas in Ethiopia.

**Table 1:** Geographic description of surveyed districts in southwestern Ethiopia

**Zone**	**District**	**Altitude (m.a.s.l.)**	**Latitude (N)**	**Longitude (E)**
Jimma	Dedo	1936-2014	07°35’-07°59’	36°43’-37°88’
	Omo nada	1728-1887	07°66’-07°62’	37°24’-37°26’
	Sokoru	1100-1600	07^o^43’-08^o^47’	37^o^19’-37^o^27’
Buno Bedele	Gachi	1630-2150	08°30’-08°41’	36°43’-36°50’
	Didessa	1873-2107	08°07’-08°15’	36°45’-36°67’
	Bedele	1863-1971	08°29’-08°53’	36°22’-36°63’
Ilu Ababora	Nopha	1500-1766	08°36’-08°41’	35°56’-35°65’
	Hurumu	1672-1830	08°29’ -08°35’	35°62’-35°73’
	Matu	1661-1747	08°24’-08°33’	35°47’-35°60’

### Sample collection

A purposive multi-stage sampling method was used to select maize-producing zones, districts within zone, and peasant associations (PA) within districts. Potential maize-producing districts and PAs were selected by consulting zone and district Agricultural and Natural Resource Offices. Subsistence farmers’ fields within each PA were randomly selected for sampling, and 1 kg per grain sample was collected from each field. The collected fresh grains were homogenized by thoroughly mixing them repeatedly and kept separately in plastic bags at 4 °C for mycological and eventual mycotoxin analyses.

### Mycological analyses

An agar plate method was used to determine the number of infected kernels and type of fungi from the maize samples. From each sample lot, 30 maize kernels were randomly selected and surface sterilized with 1% sodium hypochlorite for 1 min, rinsed three times in sterile distilled water for 30 s, and dried aseptically. From each surface disinfected sample, 10 randomly drawn kernels were placed aseptically per Petri plate (9 cm in diameter) containing potato dextrose agar (PDA) (Eur Pharm, Madrid Spain). The plates were incubated at 25 ± 2 °C for 7 days. Emerging fungal colonies were purified on new PDA and identified morphologically according to Barnett and Hunter (1987), Domsch et al. (1993), and Leslie and Summerell (2006) where applicable.

To confirm the identities of the fungal species, isolates of the *Aspergillus* genera were grown on Czapek Dox Agar (CZDA), Malt Extract Agar (MEA), and Czapek Yeast Extract Agar (CYA) at 25 °C for 7 days (Klich 2002). *Fusarium* genera were grown on Spezieller Nahrstoffarmer Agar (SNA) at 25 °C for 7 days exposed to a 12:12-h light/dark regime and identified to species level (Leslie and Summerell 2006). The two genera were isolated and identified to species level based on the micro- and macro-morphological characteristics of the standard identification keys (Klich 2002; Leslie and Summerell 2006; Pitt and Hocking 2009; Nyongesa et al. 2015).

The fungal incidence and frequency of occurrence on maize kernels were calculated as follows:

$$\mathrm{Fungi\;incidence\;on\;kernel\;}(\mathrm{\%})=\frac{\mathrm{Number\;of \;infected \;kernels}}{\mathrm{Total \;number\; of \;kernels}}\times 100$$

$$\mathrm{Frequency}\;\mathrm{of}\;\mathrm{occurrence}\;\%\;=\;\frac{\mathrm A}{\mathrm B}\times\;100$$

where *A* is the number of samples in which a particular fungus occurred and *B* is the total number of samples analyzed.

### Mycotoxin extraction and analysis

LC gradient grade methanol and glacial acetic acid (p.a.) were purchased from Merck (Darmstadt, Germany), while LC gradient grade acetonitrile and ammonium acetate (MS grade) were purchased from VWR (Leuven, Belgium) and Sigma-Aldrich (Vienna, Austria), respectively. Water was successively purified by reverse osmosis with an ELGA PURELAB Ultra analytic system from Veolia Water (Bucks, UK).

The maize grain samples were thoroughly mixed and ground through a 1 mm sieve. Then, a homogenized 5 g flour from each sample was weighed into a 50 ml polypropylene tube, and metabolites of fungi were extracted for 90 min on a GFL 3017 rotary shaker (GFL, Burgwedel, Germany) using 20 ml of extraction solvent that consisted of acetonitrile/water/acetic acid (79:20:1, v/v/v). Extracts were diluted 1:1 (v/v) with a dilution solvent (acetonitrile/water/acetic acid, 20:79:1, v/v/v), and 5 μL of each diluted extract was used for further analysis.

Liquid chromatography-tandem mass spectrometric (LC-MS/MS) analysis was conducted for the simultaneous determination of multiple microbial metabolites following Sulyok et al. (2020). Briefly, a QTrap 5500 LC-MS/MS System (Applied Biosystem, Foster City, CA, USA) equipped with a Turbo Ion Spray coupled to a 1290 Series HPLC System (Agilent, Waldbronn, Germany) was used. Chromatographic separation was carried out at 25 °C on a Gemini C18 column (150 × 4.6 mm i.d., 5 μm particle size) and a C18 4 × 3 mm i.d. security guard cartridge (Phenomenex, Torrance, CA, US). ESI-MS/MS was performed in the time-scheduled multiple reaction monitoring (MRM) mode both in positive and negative polarities in two separate chromatographic runs per sample by scanning two fragmentation reactions per analyte. Compound-specific LC-MS/MS parameters can be seen in the supplementary table of Sulyok et al. (2020).

Confirmation of positive analyte identification was obtained by the acquisition of two MRMs per analyte (with the exception of moniliformin and 3-nitropropionic acid, which exhibited only one fragmentation), which yielded 4.0 identification points in accordance with the European Commission Decision No. 2002/657 (European Commission 2002). The LC retention time and the intensity ratio of the two MRM transitions also agreed with the related values of authentic standards within 0.03 min and 30% relative intensity ratio, respectively. Quantification was performed using external calibration based on serial dilution of a multi-analyte stock solution, and results were corrected for apparent recoveries (Sulyok et al. 2020). The limits of detection (LOD) and quantification (LOQ) were determined following the EURACHEM guide based on reputability data on the lowest spiking level. The method was scrutinized in a proficiency testing scheme organized by BIPEA (Bureau Interprofessionnel d’Etudes Analytiques), International Bureau for Analytical Studies, Gennevilliers, France. The current rate of 96% of the > 2100 submitted results is in the range of − 2 < *z* <  + 2.

## Results and discussion

### Major fungi associated with maize grains in the study areas

**Fig. 2:**
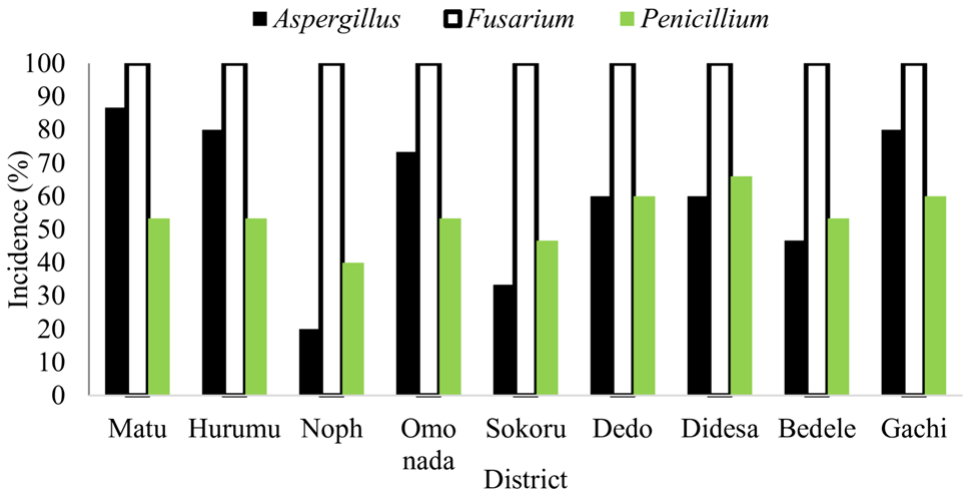
Incidence of major toxigenic fungi in fresh maize grain samples from nine districts in southwestern Ethiopia

Results of the current study revealed wider distribution of fungi across the study areas, with fungal incidences ranging between 43 and 99%. The highest mean kernel incidence was recorded at Ilu Ababora zone 88.8% followed by Buno Bedele (81.3%) and Jimma (81%). Fungal species isolated from current samples were those belonging to *Fusarium* (100%), *Aspergillus* (60%), and *Penicillium* (58%) (Fig. 2). These results agree with Binyam and Girma (2016) and Garbaba et al. (2018a, b) who reported *Aspergillus*, *Fusarium*, and *Penicillium* as the most dominant genera infecting stored maize in Jimma area. Dubale et al. (2014) also reported *Fusarium*, *Penicillium*, and *Aspergillus* from maize stored under farm storage conditions within the same area. The same genera were also reported as important storage fungi in other parts of Africa (Ekwomadu et al. 2018).

At species level, *Fusarium verticillioides* was the most frequent (99%), followed by *Fusarium graminearum* (91%), *Aspergillus niger* (66%), and *Aspergillus flavus* (47%) (data not shown). Getachew et al. (2018) and Tsehaye et al. (2017) have also reported *F. verticillioides* as the major contaminant of maize grain in Ethiopia, while Yilma et al. (2019) identified *A. flavus* as the most frequently isolated species from stored maize kernel. Results suggest that contamination of maize grains by fungi starts right on the field (pre-harvest) and continues during storage (post-harvest).

### Mycotoxins and other fungal metabolites in maize grains in the study areas

A total of 164 fungal metabolites were present in maize grain samples collected from southwestern Ethiopia at levels higher than the limits of detection, suggesting a 29% increase from the year 2015 (Getachew et al. 2018). This might have been caused by recent changes in environmental conditions favoring mycotoxin contamination. The fresh grain samples in the current study may also have higher moisture content than the stored grains creating favorable conditions for microbial activity.

The detected metabolites are grouped into 10 different categories. *Penicillium* metabolites were the most dominant ones representing 30% of the detected metabolites. They were followed by *Fusarium* metabolites that accounted for 27% of the metabolites contaminating the samples at levels higher than the limit of detection (LOD). Eighteen (11%) of the 164 metabolites were major mycotoxins and their derivatives. Unspecified metabolites, metabolites from other fungal genera, i.e., *Alternaria*, *Ascochyta*, and *Trichoderma*, and bacterial metabolites, were detected although they represented only lower proportion (1–8%) of the detected metabolites.

**Fig. 3:**
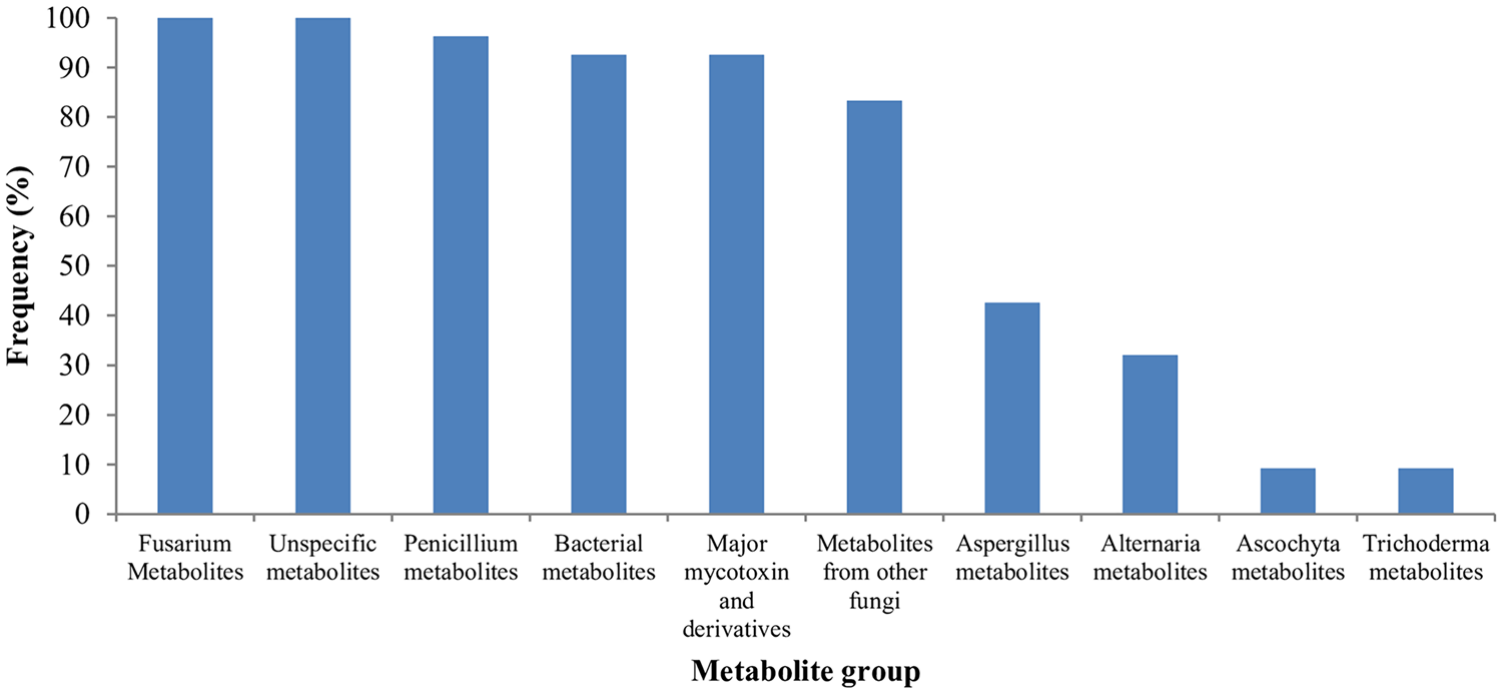
Frequency of mycotoxin groups contaminating maize grains in southwestern Ethiopia

The incidence of mycotoxin contamination also differed across the metabolite groups (Fig. 3). Accordingly, *Fusarium* metabolites and unspecified metabolites occurred in all (100%) of the samples. They were followed by *Penicillium* metabolites that were detected in 96% of the samples, and major mycotoxins and derivatives and bacterial metabolites that were present in 92% of the samples each. Getachew et al. (2018), Mesfin et al. (2022), and Mohammed et al. (2023) reported up to 26% post-harvest maize grain contamination by *Penicillium* and *Fusarium* metabolites, with *Penicillium* metabolites being the most frequently detected. *Penicillium* metabolites were also recorded at high prevalence in stored sorghum grains from Ethiopia (Mohammed et al. 2022). To the best of our knowledge, this is the first report on the contamination of maize grains by bacterial metabolites in Ethiopia although bacterial metabolites were detected in other cereals like sorghum and millet (Chala et al. 2014; Mohammed et al. 2022).

The frequency of occurrence of mycotoxin groups varied across the sample collection zones (Table 2). Ten metabolite groups were detected in maize grains from Jimma and Illu Ababora zones, with *Fusarium*, bacteria, and unspecified metabolites being the most common. The highest frequency of major mycotoxin and their derivatives were recorded in Buno Bedele (100%) followed by Ilu Ababora zone (94%), while *Fusarium* and unspecified metabolites occurred in all maize grain samples across the three zones. Maize samples were collected from areas that represent different altitude ranges (1600 to above 2000 m.a.s.l.). Major mycotoxins and derivatives were detected at altitudes ≥ 1800 m.a.s.l., but their incidence varied across altitude gradients. Eighty percent of the samples were contaminated by major mycotoxins and derivatives in low altitude areas, while the incidence of these mycotoxins increased with altitudes and levels up at around 2200 m.a.s.l.

**Table 2:** Frequency (%) of mycotoxin groups across zones of southwestern Ethiopia (*N* = 18 samples/zone)

**Mycotoxin group**	**Zone**			**Overall frequency %**
	Jimma	Buno Bedele	Illu Ababora	
Major mycotoxin and derivatives	83.3	100	94.4	14
*Fusarium* metabolites	100	100	100	13
*Aspergillus* metabolites	44.4	44.4	38.9	16
*Penicillium* metabolites	94.4	94.4	94.4	31
*Alternaria* metabolites	77.8	33.3	66.7	3
*Trichoderma* metabolites	11.1	16.7	0	3
*Ascochyta* metabolites	16.7	11.1	0	4
Metabolites from other fungi	83.3	94.4	72.2	7
Bacterial metabolites	100	83.3	94.4	1
Unspecified metabolites	100	100	100	8

### Major mycotoxins and derivatives

A total of 18 major mycotoxins and derivatives contaminated the current maize grain samples (Table 3). All the maize grain samples analyzed in the current study were contaminated by one or more of these toxins. All but one (citrinin) of the major mycotoxins and derivatives were of *Fusarium* origin. Citrinin was present only in a sample from Dedesa district of Southwest Ethiopia, at concentration of 88.7 µg/kg. This metabolite can be produced by *Aspergillus* and *Penicillium* genera (Kamle et al. 2022). Current results are in line with the findings of the mycological analysis in which *Fusarium* spp. were among the most dominant fungi contaminating the grain samples. The present findings also agree with that of Getachew et al. (2018) and Tebele et al. (2020), who reported 100% grain contamination of stored maize with mycotoxins although current samples were fresh harvests. Worldwide food crops are contaminated by mycotoxins at a prevalence of up to 80% (Eskola et al. 2020).

**Table 3:** Major mycotoxins and derivatives detected in maize grain samples collected from southwestern Ethiopia (*N* = 54 samples)

**Mycotoxin**	**% positive samples**	**Concentration (µg/kg)**		
		Mean	Median	Maximum
Citrinin	1.85	88.7	88.7	88.7
Fumonisin B1	40.7	933	104	6770
Fumonisin B2	25.9	385	161	1830
Fumonisin B3	22.2	212	116	808
Fumonisin A1	16.7	32.6	25.8	128
Deoxynivalenol	31.5	1612	814	5140
DON-3-glucoside	31.5	200	258	583
15-Acetyldeoxynivalenol	20.4	88.4	52	243
3-Acetyldeoxynivalenol	9.26	34.3	26.3	63.1
Nivalenol	63	1075	3049	17,300
Nivalenol glucoside	31.5	105	37.6	825
Fusarenon-X	18.6	61.7	52.9	149
Zearalenone	74.1	154	17.8	1310
Alpha-zearalenol	24.1	6	3.55	16.2
Beta-zearalenol	24.1	22.9	8.43	132
Zearalenone-sulfate^a^	44.4			
Monoacetoxyscirpenol	9.26	9.96	9.14	20.5
Diacetoxyscirpenol	3.7	7.59	7.59	14.4

^a^There is no standard for zearalenone-sulfate, and as such, it was not possible to quantify this metabolite at present

Zearalenone was the most prevalent major mycotoxin, occurring in 74% of the samples at average concentration of 154 µg/kg. Five maize grain samples contained zearalenone at levels ranging from 395 to 1310 µg/kg, which are above the maximum EU tolerable level (350 µg/kg) (European Commission 2007). Getachew et al. (2018) and Mohammed et al. (2023) also reported zearalenone to be the most prevalent in stored maize from Ethiopia with concentration up to 1656 and 3750 µg/kg, respectively, although at relatively higher concentrations than those in the current study. This is not surprising as those found in the current study were recovered from fresh harvests. This is because zearalenone contamination occurs both at harvest and during storage (Ropejko and Twaruzek 2021). Pleadin et al. (2012) also recorded high prevalence of same mycotoxin from harvested maize, but concentrations (max. 5.11 µg/kg) were much lower than those reported herein.

Nivalenol, another *Fusarium* mycotoxin, was the second most frequently detected mycotoxin with 63% prevalence and mean concentration of 1075 µg/kg recorded. Getachew et al. (2018) reported a 47% prevalence and maximum concentration of 793 µg/kg of this mycotoxin from stored maize in Ethiopia. Current results also relate to a report from Brazil in which the toxin was detected in 75.5% of samples at a mean concentration of 256 µg/kg (Oliveira et al. 2017). Nivalenol, nivalenol glucoside, and fusarenon-X metabolite were detected in current maize grain samples with high levels up to 17,300, 825, and 149 µg/kg, respectively.

In the current study, deoxynivalenol was detected in 31% of the samples at levels between 15.9 and 5140 µg/kg. Contamination of grains by this toxin was also reported by Xing et al. (2017) both in pre-nature drying maize (50.7 to 776.6 μg/kg) and post-nature drying maize (5.8 to 9843.3 μg/kg). Ayalew (2010) reported 5% stored maize grain contamination with levels of up to 700 µg/kg, while Getachew et al. (2018) and Mesfin et al. (2022) reported, respectively, 42 and 29% stored maize grain contamination by the same mycotoxin at levels of up to 221 µg/kg. The findings in these studies suggest a 6- to eightfold increase in DON contamination of maize grains over the past decade.

Four types of fumonisins (B1, B2, B3, and A1) were detected in the present study. Fumonisin B1 was the most frequently detected of this group occurring in 41% of maize grain samples at levels ranging from 9.46 to 6770 µg/kg. Similar prevalence of Fumonisin B1 was recorded in stored maize samples in Kenya (overall 38%) and Victoria region of Kenya (53%) (Kagot et al. 2022). Mohammed et al. (2022) reported FB1 in all maize grain samples from Ethiopia with levels ranging between 22.6 and 1058 µg/kg in stored maize. In North China, all 44 maize samples collected during pre- and post-nature drying periods were contaminated by FB1 with a mean level of 133 µg/kg (Xing et al. 2017). FB2, FB3, and FA1 were detected in 17–26% of samples at 385, 212, and 32.6 µg/kg, respectively. The present findings confirmed the widespread contamination of maize by *Fusarium* metabolites across locations and altitude gradients. Our findings agree with previous reports on these mycotoxins from Ethiopia (Getachew et al. 2018; Mesfin et al. 2022; Mohammed et al. 2023; Tsehaye et al. 2017; Worku et al. 2019). However, it should be noted that the findings reported in our study were from fresh harvests, while the previous studies were on stored maize.

### Miscellaneous *Fusarium* metabolites

Bikaverin was the most prevalent *Fusarium* metabolite, occurring in 98% of the samples followed by moniliformin and aurofusarin (96% each) (Table 4) being recovered from samples at mean concentrations of 61.8, 1006, and 4262 µg/kg, respectively. Relatively high prevalence of these *Fusarium* metabolites was also reported from northern Serbia in two consecutive years (Radic et al. 2021). Moniliformin is considered a leading emerging toxin in South Africa with a high prevalence of 98% at a maximum contamination level of 1130 µg/kg (Ekwomadu et al. 2020). The presence of this mycotoxin at unusually higher levels in samples reported herein may confirm its increasing importance in Sub-Saharan Africa. A metabolite produced by *Fusarium* spp. (Nihei et al. 1998; Ezekiel et al. 2020), W493B was also present in 22% of the samples at higher levels (mean 162 µg/kg; max. 652 µg/kg).

**Table 4:** *Fusarium* metabolites other than major mycotoxins and derivatives contaminating maize grains in southwestern Ethiopia (*N* = 54 samples)

**Mycotoxin**	**% positive samples**	**Concentration (µg/kg)**		
		Mean	Median	Maximum
Moniliformin	96.3	1006	675	4410
Beauvericin	92.6	187	22.9	1550
Beauvericin A	64.8	4.09	0.75	33.8
Culmorin	51.9	22.4	16.1	104
15-Hydroxyculmorin	63	497	249	3960
5-Hydroxyculmorin	16.7	1739	1290	5150
Acuminatum B	14.8	102	50.2	303
Acuminatum C	5.56	75.6	70.3	129
Apicidin	22.2	76.8	16.3	490
Apicidin C	3.70	3.17	3.17	3.61
Apicidin D2	16. 7	13.7	7.28	72
Aurofusarin	96.3	4262	628	57,500
Bikaverin	98.2	61.8	20.6	594
Butenolid	42.6	81.2	56.4	263
Chrysogin	20.4	12.8	9.48	37.8
Deoxyfusapyron	61.1	134	54.4	800
Equisetin	5.56	3.66	3.26	5.88
Epiequisetin	5.56	0.79	0.66	1.16
Fusaproliferin	61.1	2178	938	20,200
Siccanol	59.3	3602	1285	39,500
Fusapyron	75.9	131	29.6	1430
Fusaric acid	92.6	725	189.5	9280
Fusarin C	7.41	332	283	626
Gibberellic acid	11.1	12.6	9.17	34.1
Gibepyron D	35.2	70.8	35.2	314
Sambucinol	3.7	47	47	49.7
Sambutoxin	64.8	0.97	0.35	8.61
W 493B	22.2	162	96.1	652

### *Aspergillus* metabolites

None of the current maize grain samples were contaminated by aflatoxins and ochratoxins. However, 21 metabolites from aflatoxin/sterigmatocystin pathway were detected in the current study (Table 5). Among them, averufin and sterigmatocystin were the most prevalent with 26 and 24% frequencies and mean concentrations of 0.46 and 2.49 µg/kg, respectively. However, aflatoxins and ochratoxins have been previously reported in stored maize at varying levels (Ayalew 2010; Chauhan et al. 2016; Getachew et al. 2018; Worku et al. 2019). The presence of metabolites from aflatoxin/sterigmatocystin pathway suggests the likely contamination of the grains with *Aspergillus versicolor* (Pitt 2014) or contamination by other fungal species that might have interfered with the biosynthesis of aflatoxins at the sterigmatocystin step. Additionally, the absence of aflatoxins in the current samples could have been caused by lower temperatures that typically prevail in Ethiopia during October–December.

**Table 5:** *Aspergillus* metabolites present in maize grain samples (*N* = 54 samples)

**Mycotoxin**	**% positive samples**	**Concentration (µg/kg)**		
		Mean	Median	Maximum
Sterigmatocystin	24.1	2.49	0.58	18.6
Methoxysterigmatocystin	1.85	1.83	1.83	1.83
Versicolorin A	1.85	1.54	1.54	1.54
Versicolorin C	11.1	0.32	0.2	1.02
Versiconol	7.41	2.05	1	5.86
Averantinmethylether	9.26	0.26	0.2	0.46
Averufin	25.9	0.46	0.32	3.03
Norsolorinic acid	1.85	1.71	1.71	1.71
Seco-sterigmatocystin	9.26	0.43	0.36	0.94
3-Nitropropionic acid	5.56	5.71	5.65	7.98
Asperflavine	5.56	2.43	2.4	2.78
Aspulvinone E^a^	5.56			
Cytochalasin E	16. 7	6.53	3.2	25.9
Demethylsulochrin	3.7	2.37	2.37	2.55
Deoxytryptoquialanine^a^	5.56			
Nortryptoquialanine^a^	3.7			
Tryptoquialanine^a^	3.7			
Fumiquinazoline derivative	3.7	107	107	115
Lecanoric acid	81.5	26.7	27.6	75.2
Pseurotin A	1.85	45.5	45.5	45.5
Vermistatin	3.7	1.47	1.47	1.49

^a^There were no standards for these toxins; hence, it was not possible to quantify them at present

### *Penicillium* metabolites

Questiomycin derivative, pestalotin, and 7-hydroxypestalotin were the most prevalent *Penicillium* metabolites contaminating 94, 89, and 87% of the maize grain samples, occurring at mean concentrations of 185, 27.2, and 36.6 µg/kg, respectively (Table 6). The highest frequency of occurrence of those three metabolites on freshly harvested maize was in line with a previous study on post-harvest maize by Mohammed et al. (2023). However, their levels were about 30% higher than previously reported. Rugulovasine A was also detected at higher prevalence (74%) and concentration (mean 241 but up to 3420 µg/kg) compared to earlier reports of 33% prevalence and a maximum concentration of 1159 µg/kg (Getachew et al. 2018).

**Table 6:** *Penicillium* metabolites contaminating maize grains in southwestern Ethiopia. (*N* = 54 samples)

**Mycotoxin**	**% positive samples**	**Concentration (µg/kg)**		
		Mean	Median	Maximum
Mycophenolic acid	9.26	267.4	16.8	161
Mycophenolic acid IV	1.85	30	30	30
O-Desmethyl-mycophenolic acid	1.85	7.68	7.68	7.68
3-Hydroxymitorubin	3.7	4942	4942	9870
Mitorubin	11.1	354	3.25	2110
Pestalotin	88.9	27.2	6.13	513
7-Hydroxypestalotin	87	36.6	11.4	592
Agroclavin	5.56	2.71	0.38	7.45
Chanoclavin	51.9	5.25	0.35	71.2
Festuclavine	3.7	3	3	5.76
Andrastin A	9.26	11.1	5.39	29.4
Andrastin B	3.7	40.9	40.9	46.9
Griseofulvin	14.8	125	9.26	558
Griseophenone A	1.85	3.92	3.92	3.92
Griseophenone B	7.41	879	583	2290
Griseophenone C	7.41	28.8	17.7	75.4
Dechlorogriseofulvin	11.1	52.4	23.8	168
Dehydrogriseofulvin	1.85	3.01	3.01	3.01
Demethylgriseofulvin	1.85	2.17	2.17	2.17
Barceloneic acid	9.26	25.4	14.7	82.7
Berkeleyamide B	7.41	5.66	3.11	14.3
Berkeleyamide C	12.96	0.33	0.17	1.21
Bilaid A	14.8	3.48	2.59	10.7
Cyclopenin	13	2.09	0.96	6.48
Cyclopenol	9.26	29.4	9.05	85.7
Cyclopeptine	7.41	4.45	2.74	12
Flavoglaucin	13	118	14	417
Ergine	1.85	2.12	2.12	2.12
F01 1358-A	1.85	2.73	2.73	2.73
NP1243	5.56	31.9	26.2	51
NP139	13	154	136	556
O-Methylviridicatin	1.85	0.21	0.21	0.21
Penitrem A	3.7	8.76	8.76	10.7
PF 1163A	1.85	0.48	0.48	0.48
Pinselin	1.85	2.61	2.61	2.61
Questiomycin	1.85	1.56	1.56	1.56
Questiomycin Derivative	94.4	185	27.3	3250
Quinolactacin A	12.96	0.13	0.08	0.34
Roquefortine C	11.1	1.81	1.02	5.4
Roquefortine D	1.85	8.79	8.79	8.79
Rugulovasine A	74.1	241	21.1	3420
Scalusamid A	1.85	0.35	0.35	0.35
Sclerotinin A	18.5	45.7	4.65	370
Terragine^a^	1.85			
Verrucofortine	7.41	0.93	0.11	3.45
Verrucosidin^a^	1.85			
Viridicatol	7.41	35.3	34.9	47.9
Viridicatum toxin	9.26	2509	935	9870

^a^There are no standards for these toxins and hence it was not possible to quantify them at present

### Metabolites of other fungi

Maize grain samples analyzed in the current study were contaminated by metabolites produced by a diverse range of fungi in addition to those of the *Fusarium*, *Penicillium*, and *Aspergillus* genera (Table 7). Macrosporin, as an example, was the most frequently detected mycotoxin of *Alternaria* contaminating 56% of the samples at a mean level of 11.6 µg/kg. *Trichoderma* and *Ascochyta* metabolites were also detected but at a low frequency of 2–6%. Additional 12 metabolites were found contaminating the current maize sample at frequencies ranging from 1.85 to 53.7% (Table 7). These included radicicol and abscisic acid that contaminated 53.7 and 29.6% of the samples at mean concentrations of 143 and 973 µg/kg, respectively.

**Table 7:** *Alternaria*, *Trichoderma*, *Ascochyta*, and additional fungal metabolites detected in maize grain samples collected from southwestern Ethiopia (*N* = 54 samples)

**Group**	**Mycotoxin**	**% positive samples**	**Concentration (µg/kg)**		
			Mean	Median	Maximum
*Alternaria* metabolites	Tenuazonic acid	22.2	1044	58.8	5930
	Alternariol	1.85	82.5	82.5	82.5
	Alternariolmethylether	7.41	41.5	0.86	164
	4-Hydroxyalternariol	1.85	33.1	33.1	33.1
	Altersetin	1.85	26.8	26.8	26.8
	Altersolanol^a^	16.7	-	-	-
	Altertoxin-I	3.70	11.0	11.0	17.7
	Macrosporin	55.6	11.6	1.41	153
*Trichoderma* metabolites	Trichodermin	3.70	8.23	8.23	8.43
	Trichodimerol	3.70	3.77	3.77	5.00
	Dihydrotrichotetronine	3.70	60.5	60.5	97.1
	Trichotetronine	5.56	79.3	63.4	135
	Sorbimycin^a^	3.7	-	-	-
*Ascochyta* metabolites	Ascochlorin	1.85	0.35	0.35	0.35
	Ilicicolin A	3.7	1.51	1.51	2.12
	Ilicicolin B	3.7	2.62	2.62	3.09
	Ilicicolin H	3.7	14.1	20.9	14.1
Metabolites from other fungi	Abscisic acid	29.6	973	378	5990
	Calphostin C	5.56	941	94.4	160
	Chloromonilic acid B	3.7	2.99	2.99	3.52
	Cladosporin	5.56	22.5	8.61	53.9
	Clonostachydiol	1.85	2.10	2.10	2.10
	CRM 646A	18.5	156	31.5	1100
	Dichlordiaportin	9.26	40.1	23.9	120
	Diplodiatoxin	3.7	7708	7708	15,400
	Ergometrinine	1.85	0.58	0.58	0.58
	Moniliphenone	11.1	1.16	0.92	2.97
	Monocerin	5.56	29.8	1.40	87.4
	Pyrrocidine A	18.5	32.1	14.1	126
	Radicicol	53.7	143	46.7	822

^a^No standards were available for these metabolites and hence the metabolites could not be quantified

## Conclusion

The present study shows that all the maize grains collected from farmers’ fields of southwestern Ethiopia were contaminated with a multitude of fungi and mycotoxins. The fungi genera *Fusarium*, *Aspergillus*, and *Penicillium* were the major contaminants of maize grain in the study area at harvest. Unlike previous studies that focused on post-harvest maize, the current work demonstrated the widespread contamination of maize at harvest stage. The presence of multiple fungi and associated mycotoxins, as reported at levels higher than international standards in this study, raises serious concerns about maize-based food and feed safety.

Therefore, special attention is needed to protect maize from these harmful compounds at pre- and post-harvest to ensure food security and safety in Ethiopia and enhance the export of maize grains from the country. There should be a concerted effort to quantitatively detect toxigenic fungi and associated mycotoxins across all stages of the maize value chain in the country. Additional studies should also be carried out to determine the biophysical factors that predispose maize to infection by toxigenic fungi and contamination with mycotoxins. The current work also demonstrates the need to create awareness on fungal and mycotoxin contamination along the maize value chain among the various actors. Additionally, sustainable monitoring and management strategies for mycotoxigenic fungi should be developed and put in place to improve food and feed safety and enhance trade. Harvest and post-harvest operations including timely harvesting, proper drying, sorting grains, and use of improved storage that prevents/minimizes contamination by toxigenic fungi and subsequent mycotoxin contamination should also be given due attention to improve grain quality and safety and protect consumers’ health and social resilience of local communities.

## Data Availability

Data supporting the findings of this study are available within the paper. Additional data can be provided by the corresponding author upon reasonable request.
